# Confronting two biomolecular techniques to detect *NRF2* gene polymorphism biomarkers

**DOI:** 10.4155/fsoa-2018-0075

**Published:** 2018-12-11

**Authors:** Pieranna Chiarella, Renata Sisto, Ario de Marco

**Affiliations:** 1 INAIL Research - Department of Occupational & Environmental Medicine, Epidemiology & Hygiene. Via Fontana Candida 1 – 00078 Monte Porzio Catone (RM), Italy; 2 Laboratory for Environmental & Life Sciences - University of Nova Gorica, Vipavska 13, S-5000 Nova Gorica, Slovenia

**Keywords:** biomarker, confronting two-pair primers, gene polymorphism, *NRF2*, oxidative stress, PCR, RFLP, susceptibility

## Abstract

**Aim:**

Gene polymorphism biomarkers identify individual susceptibility to environmental and occupational hazards. The conventional approach considers polymerase chain reaction (PCR) followed by restriction fragment length polymorphism analysis (RFLP), a reliable but expensive and time-consuming two-step procedure. Therefore we evaluated the simpler method confronting two-pair primers (CTPP)–PCR for its robustness and applicability to epidemiologic studies.

**Materials & methods:**

We compared CTPP–PCR and PCR–RFLP techniques to detect two *NRF2* polymorphisms in a set of biological samples.

**Results:**

CTPP–PCR produced contradictory results and required the orthogonal technique for confirming the data.

**Conclusion:**

In contrast to PCR-RFLP, CTPP–PCR of *NRF2* polymorphisms resulted in ambiguous genotyping which strongly jeopardized heterozygosis classification. The necessity of long optimization and control procedures nullified the potential advantages of CTPP–PCR in terms of costs and time.

## Lay abstract

Confronting two-pair primers-polymerase chain reaction (CTPP–PCR) is potentially a fast, simple and cost-efficient method for the detection of gene polymorphism biomarkers. This technique was used to genotype two SNPs of *NRF2* gene in a set of biological samples but resulted in ambiguous results. Therefore, it was necessary to validate the CTPP–PCR genotyping data with the orthogonal technique PCR-RFLP. Since reproducibility is a critical issue in both biomedical research and diagnostics, we advise pairing CTPP–PCR with control methods and evaluating whether the overall package is still convenient for massive genetic screening.

## Graphical abstract

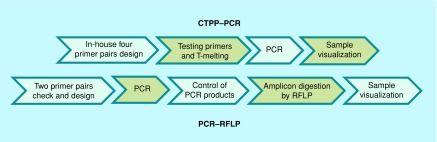

Single nucleotide polymorphisms (SNPs) are the most common type of genetic variations in humans and are evaluated as susceptibility biomarkers in studies of exposure to xenobiotics [1]. Individual genetic profiles may provide useful indications of susceptibility to toxicants and consequently improve the risk assessment procedures used to ensure health protection in occupational and environmental contexts [2].

Polymerase chain reaction (PCR) followed by restriction fragment length polymorphism (RFLP) still remains the gold standard method to identify SNPs [3] and despite the appearance of high-throughput techniques in the last decade (i.e., denaturing high-performance liquid chromatography, high-resolution melting analysis, SNaPshot, next-generation sequencing) [4,5], it remains the preferred option for genotyping in laboratories that cannot afford expensive instrumentations or have no access to genotyping service. In this two-step process, genes are amplified with a primer pair and afterward the amplicon is digested by specific endonuclease restriction enzymes before final evaluation of the digestion patterns. PCR with confronting two-pair primers (CTPP–PCR) has been developed as a faster, simpler and inexpensive alternative to PCR-RFLP. The original method, conceived by Hamajima and coworkers [6], has been further improved and adapted to an increasing panel of SNPs in the last 15 years [7–10]. The rationale is based on the design of two couples of primers which amplify: the wild-type allele (F1-Forward 1 and R1-Reverse 1); the variant allele (F2-Forward 2 and R2-Reverse 2); and any allele (F1 and R2). In both F2 and R1 sequences the nucleotide located at the 3′ end is modified to allow the amplification of the specific SNP [6]. The PCR reaction results in two or three amplicons of different length, depending on the individual haplotype. F1-R1 and F1-R2 are specific for the homozygous wild-type allele; F1-R2 and F2-R2 are specific for the homozygous variant allele; F1-R1, F2-R2, F1-R2 characterize the heterozygous genotype. Amplicons are visualized on agarose gel by electrophoresis without further steps. However, since two forward and two reverse primers are used simultaneously in the same reaction, competition among oligonucleotides may occur and the stronger affinity of the most specific may result in preventing the other from binding to the template [11,12]. This condition might critically jeopardize the final result [13].

Based on previous published data [6–9], we applied CTPP–PCR to the screening of selected SNPs of *NRF2* (Gene ID: 4780). This gene is important in the study of individual exposure to toxicants and carcinogens being a key regulator of the cell transcriptional response to oxidative stress [14]. Studies on animal models have shown that *NRF2* protects the inner ear against age-related hearing injuries and gentamicin ototoxicity by up-regulating antioxidant enzymes and detoxifying proteins [15]. In a cohort subjected to occupational noise exposure, *NRF2* rs6721961 SNP accounted for the increase of susceptibility to noise-induced hearing loss caused by free radical production [16]. Other studies showed that *NRF2* SNPs of the promoter region have been associated with vitiligo risk in the Chinese population [17,18]. This genetic disorder is often under-estimated even though the severity of epidermal lesions tends to worsen when individuals are occupationally exposed to chemical and physical stressful agents.

At least 17 *NRF2* SNPs have been identified for their association with disease risk and evaluated for their effects on gene expression and function [14]. In particular, three polymorphisms (namely rs6721961, rs35652124 and rs6706649) located in the 5′ promoter region of *NRF2* seem to be associated to specific susceptibilities and were proposed as biomarkers [16–20].

Since unambiguous and reproducible results are essential requests in diagnostics, we wanted to compare the robustness and reliability of CTPP–PCR with PCR-RFLP for the characterization of two of these polymorphisms, *NRF2* rs6721961 (-617 C/A) and *NRF2* rs35652124 (-653 A/G), with the idea to extend its application to the study of larger populations of individuals exposed to risk factors.

## Materials & methods

### Human participants to the study

16 Caucasian individuals, belonging to our Institute, were enrolled on the basis of their personal agreement as volunteers to participate to the study. A second group of eight volunteers was enrolled from a biomonitoring campaign directed to workers employed in a ship construction company in Tuscany (Italy). Of these, four were ship painters and the others were administrative personnel. All individuals were eligible and agreed to the methodological study after giving their informed consent. Inclusion criteria were age 30–55 years; participants included both males and females. Data obtained in this study were anonymous and can only be interpreted on a population level. All procedures performed in this study involving human participants (Declaration of Helsinki) were in accordance with the ethical standards of our institutional committee and in accordance with the local ethics committee (USL North-West, Tuscany, July 2017).

### DNA extraction

Genomic DNA was isolated from urine exfoliate and blood of the recruited human volunteers. DNA was extracted from urine exfoliate according to the method described previously [21] whereas blood DNA was recovered by using the QiAmp DNA blood mini kit cat. N. 51306 (Qiagen, Germany). Blood was sampled from medical staff with informed consent and according to the INAIL ethical guidelines.

### Confronting two-pair primers polymerase chain reaction

Confronting two-pair primer sequences specific for the -617 C/A and -653 A/G polymorphisms of *NRF2* (Table 1A) and experimental conditions were designed according to previous publications [19,22]. Oligonucleotides were checked for correct DNA complementarity by publicly available softwares Kalign and Multalin (www.ebi.ac.uk/Tools/msa/kalign/; multalin.toulouse.inra.fr/multalin/) and purchased by Metabion GmbH – Dasit Carlo Erba (Germany-Italy).

**Table 1A.** **Primer sequences and melting temperatures.**

**SNP**	**Nucleotide sequence**	**T melting**
rs6721961		

F1	CTCCGTTTGCCTTTGACGAC	56.6°C

R1	GGGGAGATGTGGACAGC**G**	58.3°C

F2	GCGAACACGAGCTGCCGG**A**	63.3°C

R2	CCCTGATTTGGAGTTGCAGAACC	58.3°C

rs35652124		

F1	GGGGTTCCCGTTTTTCTCCC	58.5°C

R1	GCAGTCACCCTGAACGCCC**T**	62.5°C

F2	GACACGTGGGAGTTCAGAGG**G**	59.6°C

R2	CTTTTATCTCACTTTACCGCCCGAG	57.4°C

F2 and R1 allele-specific nucleotides at 3′ end are highlighted in bold.

SNP: Single nucleotide polymorphism.

PCR product details and experimental PCR conditions are summarized in Table 1B and Table 1C, respectively. Since F2 (rs6721961) and R1 (rs35652124) have melting temperature significantly different from the others, CTPP–PCR reactions were performed using the gradient option of the thermocycler (Multigene optimax thermal cycler, Aurogene SRL, Italy) that enables contemporary polymerization reaction at two different annealing temperatures (T_a_). All PCRs were performed with 1 unit/sample of Kapa Hot Start Taq polymerase, Biosystems Cat. KK1508 (Sigma-Aldrich, MO, USA) except for the PCR shown in Figure 3 where 2.5 units/sample of AmpliTaq Gold polymerase, Applied Biosystems Cat. N8080161 (ThermoFisher Scientific, MA, USA) were used. PCR products were separated on 1.5% agarose gel Cat. BMR 918100 (Euroclone, MI, Italy) with TBE (Tris, Boric acid, EDTA) buffer and stained with gel red staining solution (Biotium, CA, USA). Gel images were acquired by means of PXi4 image analyzer equipped with the GenSys software (Syngene, MD, USA).

**Table 1B.** **Confronting two-pair primers-polymerase chain reaction and  restriction fragment length polymorphism product length.**

**Genotype**	**rs6721961 C/A Product size (CTPP–PCR)**	**rs35652124 A/G Product size (CTPP–PCR)**	**rs6721961 C/A Product size (PCR–RFLP)**	**rs35652124 A/G Product size (PCR–RFLP)**
Homozygous (wild-type) alleles	282&113 bp (CC)	318&146 bp (AA)	191&91 bp	180&138 bp

Heterozygous alleles	282&205&113 bp (CA)	318,213,&146 bp (AG)	282&191&91 bp	318&180&138 bp

Homozygous (variant) alleles	282&205 bp (AA)	318&213 bp (AA)	282 bp (AA)	318 bp (GG)

CTPP: Confronting two-pair primer; PCR: Polymerase chain reaction; RFLP: Restriction fragment length polymorphism.

**Table 1C.** **Conditions used for confronting two-pair primers–polymerase chain reaction amplification.**

**SNP**	**rs6721961 C/A**	**rs35652124 A/G**
Number of cycles	35	35

Hot start	95°C, 5 min	95°C, 5 min

Denaturation	95°C, 1 min	95°C, 1 min

Annealing	58°C/65°C, 1 min	59°C/66°C, 1 min

Extension	72°C, 1 min	72°C, 1 min

Final extension	72°C, 5 min	72°C, 5 min

End of reaction	4°C, ∞	4°C, ∞

SNP: Single nucleotide polymorphism.

### Polymerase chain reaction-restriction fragment length polymorphism

*NRF2* -617C/A and *NRF2* -653A/G amplicons were obtained using primers F1 and R2 for each SNP, as indicated in Table 1A and at the same experimental conditions used in CTPP–PCR and with the same Taq polymerase except for the T_a_, which was 62°C. The expected amplicon size was 282 bp for *NRF2* -617 C/A and 318 bp for *NRF2* -653 A/G (Supplementary Figure 1). Enzymatic digestion of *NRF2* -617 C/A amplicon was performed with 7.5 units per sample of NgoMIV restriction enzyme (New England Biolabs, MA, USA; Euroclone), incubated for 90 min at 37°C without enzyme inactivation. Enzymatic digestion of *NRF2* -617 A/G amplicon was performed with 15 Units per sample of BseRI restriction enzyme (New England Biolabs, Euroclone), incubated for 90 min at 37°C followed by 20 min at 80°C for enzyme inactivation. The restriction enzymes were a kind gift from EMBL. The predicted products obtained by RFLP have been worked-out and verified using the following bioinformatics simulators: www.bioinformatics.org/sms2/rest_digest.html and www.restrictionmapper.org/ and are shown in Table 1B.

## Results

**Figure 1.**
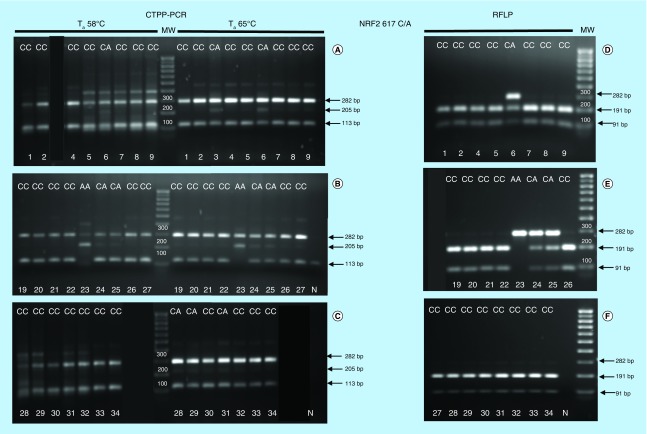
**Comparison between CTPP–PCR and RFLP results for *NRF2*-617C/A SNP.** Confronting two-pair primers–polymerase chain reaction of *NRF2* rs6721961 (-617 C/A) single nucleotide polymorphism performed at two different T_a_ (58°- 65°C) using 1 unit/sample of Kapa Hot Start Taq polymerase **(A–C)**. Restriction fragment length polymorphism analysis after amplicon digestion with NgoMIV **(D–F).** Sample ID and genotype are indicated by numbers and letters at the top and bottom of figures. CTPP: Confronting two-pair primer; N: Negative control indicates DNA template omission in the polymerase chain reaction; MW: Molecular weights (100 bp up to 1000 bp); RFLP: Restriction fragment length polymorphism; SNP: Single nucleotide polymorphism. The black lanes are irrelevant samples for which the corresponding confronting two-pair primers–polymerase chain reaction was not reported.

**Figure 2.**
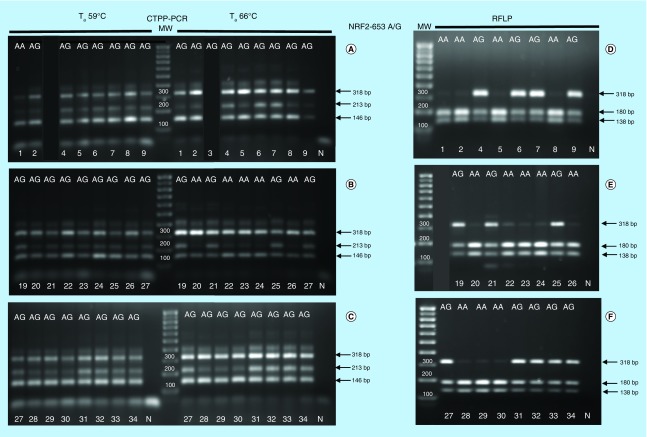
**Comparison between CTPPCR and RFLP results for *NRF2*-653A/G.** Confronting two-pair primers–polymerase chain reaction of *NRF2* rs35652124 (-653 A/G) single nucleotide polymorphism performed at two different T_a_ (59°- 66°C) using 1 unit/sample of Kapa Hot Start Taq polymerase **(A–C)**. Restriction fragment length polymorphism analysis after amplicon digestion with BseRI **(D–F)**. Sample ID and genotype are indicated by numbers and letters at the top and bottom of figures. N: Negative control indicates DNA template omission in the polymerase chain reaction; MW: Molecular weights (100 bp up to 1000 bp). The black lanes are irrelevant samples for which the corresponding confronting two-pair primers–polymerase chain reaction was not reported.

*NRF2* -617C/A and *NRF2* -653 A/G SNP were characterized in 24 individuals by initially using already optimized experimental conditions [19,23]. The most critical parameter of CTPP–PCR is the competition of the primers for the target DNA. Since the annealing temperature (T_a_) regulates primer binding to complementary DNA, we performed PCR at two alternative T_a_ to identify conditions which minimize primer competition. The results of the polymorphism analysis of *NRF2* -617 C/A and -653 A/G SNPs relative to the 24 individual samples are shown in Figures 1 and 2. For -617 C/A, PCR products were in general better distinguishable when the highest T_a_ (65°C) was used. For instance, the heterozygous genotype of sample #6 was visible when the highest T_a_ (65°C) was used but was undetectable when processed at the lowest T_a_ (58°C; Figure 1A). Also the genotype of sample #24, #25 obtained by CTPP–PCR (Figure 1B) was uncertain due to the presence of an intermediate faint band. The results could be confirmed and validated only by comparison with RFLP digestion patterns (Figure 1 A, B, C vs D, E, F). Looking at CTPP–PCR results, subject #28 and #29 (Figure 1C) could be misinterpreted as heterozygous instead of being homozygous, as shown in Figure 1F. In general, for several samples, the genotypes obtained by CTPP–PCR using the two different temperatures were not coincident.

**Figure 3.**
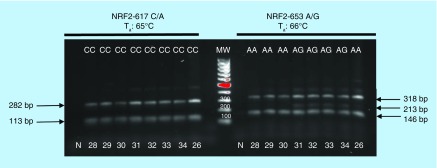
**Confronting two-pair primers–polymerase chain reaction for *NRF2* -617C/A and -653A/G was carried out at the indicated T_a_ using 2.5U/sample of AmpliTaq Gold polymerase.** N: Negative control indicates DNA template omission. MW: Molecular weights (100 bp up to 1000 bp).

Similarly, when the -653 C/A SNP was analyzed by CTPP–PCR, evident discrepancies in the genotyping results obtained at the two different T_a_ were observed (Figure 2). For instance, it was impossible to draw sure conclusions about the genotype of several subjects, such as #2, #5, #8, #20, #22, #23, 24#, #26, #28, #29, #30. The genotype validation by RFLP analysis (Figure 2D, E, F) confirmed the homozygous wild-type genotypes of all those samples.

The sensitivity of the methodology to experimental factors was further challenged by substituting the polymerase in the reaction (Figure 3). Again, the results did not correspond to the previous ones obtained by using a Taq polymerase from another commercial vendor (Table 2). In these conditions, it became impossible to assess the overall population distribution of the alleles corresponding to the -617 C/A and -653 A/G SNPs unless we could confirm the validity of results by RFLP.

**Table 2.** **Effect of T_a_ and Taq polymerase on the genotypic profile by confronting two-pair primers–polymerase chain reaction.**

**Subject ID**	**NRF2 -617C/A**		**NRF2 -653A/G**	
	**Taq KAPA Biosystems**		**Taq KAPA Biosystems**	

	**T_a_ 58°C**	**T_a_ 65°C**	**T_a_ 59°C**	**T_a_ 66°C**

#28	CC	CA	AG	AG

#29	CC	CA	AG	AG

#31	CC	CA	AG	AG

#34	CC	CC	AG	AG

	**T_a_ 65°C**		**T_a_ 66°C**	

	**TAQ Applied Biosystems**	**Taq KAPA Biosystems**	**TAQ Applied Biosystems**	**Taq KAPA Biosystems**

#26	CC	CC	AA	AA

#28	CC	CA	AA	AG

#29	CC	CA	AA	AG

#30	CC	CC	AA	AG

#31	CC	CA	AG	AG

#32	CC	CC	AG	AG

#33	CC	CC	AG	AG

#34	CC	CC	AG	AG

## Discussion

We applied CTPP–PCR to detect two polymorphisms located in the promoter of *NRF2* gene and this could be informative for epidemiologic studies on occupational and environmental health. The initial aim of this work was to validate a faster, simpler and cost-effective alternative to the conventional two-step PCR-RFLP for SNP evaluation (Table 3). However, our attempt to replicate the published protocol described in other papers [19,22,23] shows that CTPP–PCR produced rather ambiguous and even contradictory results that had to be validated by employing an orthogonal genotyping technique. Such results confirmed that CTPP–PCR is a technique extremely sensitive to experimental conditions, the most critical of which is the design of the four primers to obtain the most possible similar T_m_ values and avoiding primer pairs competition for the DNA template during PCR [13,24]. However, variations of T_a_ value and polymerase type also resulted in differing results, underlining that CTPP–PCR requires long optimization and control procedures for each single SNP. Clearly, such procedures would nullify all the potential advantages of CTPP–PCR in terms of costs and time (Table 3) with respect to PCR-RFLP [6–9,19,22].

**Table 3.** **Comparison of time and cost between confronting two-pair primers–polymerase chain reaction and polymerase chain reaction-restriction fragment length polymorphism. **

**Item**	**Cost**		**Time**	
	CTPP–PCR	PCR-RFLP	CTPP–PCR	PCR-RFLP

Primers	46.50 €	23.25 €	3 h	3 h

Shipping	6.50€	6.50€	–	–

Taq polymerase 500 U	228.00€	114.00€	–	–

RFLP enzyme *NgoMIV (10,000 U/ml)*	0.00€	55.00€	0	2 h

RFLP enzyme *BseRI (5000 U/ml)*	0.00€	60.00€	0	2 h

Sample separation on agarose gel	43.50€ (10 g)	87.00€ (20 g)	3 h (one gel for PCR)	6 h (two gels for PCR and digestion)

0,2 ml tubes	8.00€	16.00€	–	–

1 ml tubes	5.00€	10.00€	–	–

TBE buffer	30.60€	61.20€	–	–

Total	368.10€	432.95€	6 h	13 h

NgoMIV and BseRI restriction enzymes have been used for PCR-RFLP of -617C/A and -653A/G *NRF2* polymorphism in [14].

This table does not consider the costs and time necessary for the control experiments to validate the data produced by CTPP–PCR.

CTPP: Confronting two-pair primer; PCR: Polymerase chain reaction; RFLP: Restriction fragment length polymorphism.

Since in our experiments CTPP–PCR was performed according to published methods, with the same described primers and at the same experimental conditions [19,23], we cannot exclude inconsistencies in previous publications because no image of genotyping was shown in the original papers [19,22]. This condition prevents assessing if ambiguous results, such as the ubiquitous and low-intensity presence of the F2-R2 -653A/G SNP amplicon, were generated also in those experiments. In any case, diagnostic genotyping should be objective and not subjectively interpreted by researchers according to the varying intensity of PCR products. Therefore, we would suggest considering with caution CTPP–PCR for SNP analysis, limiting its use to those cases in which no reliable restriction enzyme would be available for PCR-RFLP. As a control step, we recommend to sequence the obtained fragments. More generally, we plead for the publication of reports with complete experimental dataset for comprehensive evaluation of their quality.

## Conclusion

CTPP–PCR is a potentially fast, simple and cost-saving methodology useful in detecting gene polymorphisms by using two primer pairs. However, as shown here, ambiguous genotyping might occur regardless of the optimization attempts. This strongly depends on experimental conditions, primer design and specificity, which may result in unreliable classification of genotypes, particularly for heterozygosis. The identification of the appropriate experimental conditions for several gene polymorphisms requires time, work and financial resources. Since biomedical research and diagnostics must provide high data reproducibility [25], we strongly advise accurate protocol optimization and result validation prior to use CTPP–PCR for massive genetic screening.

## Future perspective

Single nucleotide substitutions are the most common type of genetic variations in humans. These are known as biomarkers of susceptibility toward the exposure to xenobiotics and are of great relevance for public health. In the occupational setting, the association between exposure to xenobiotics and variable functionality of SNPs involved in a specific metabolism is extremely important to identify individual susceptibilities and improve worker health and safety. Besides methods such as PCR–RFLP, CTPP–PCR, PCR–ARMS (Amplification Refractory Mutation System) and mass spectrometry, further alternative technologies have been developed, in other words, Taqman assay, High-Resolution Melting and different types of mini-sequencing. It seems that the trend in the development of genotyping is moving toward multiplexing strategies and miniaturization [26] for assessing a large number of SNPs simultaneously [27]. In the field of occupational medicine, the progressive availability of data concerning the allele frequencies of the polymorphisms involved in detoxification would contribute to identify the most predictive tests, and consequently to reduce less informative genetic tests.

**Executive summary**

- Confronting two-pair primers–polymerase chain reaction is a potentially fast, simple and cost-saving method to detect gene polymorphism biomarkers based on the use of two primer pairs.
- When the technique was applied for the genotyping of two single nucleotide polymorphisms of *NRF2* gene produced ambiguous results.
- Validation of confronting two-pair primers–polymerase chain reaction with an orthogonal technique is mandatory to ensure reliable results for gene polymorphism biomarkers.
