# Supplementary figures and images for: Confronting two biomolecular techniques to detect NRF2 gene polymorphism biomarkers
Source: Future Sci OA. 2018 Dec 11;5(2):FSO361. doi: 10.4155/fsoa-2018-0075 (PMC6391633; doi:10.4155/fsoa-2018-0075)

## Slide 1
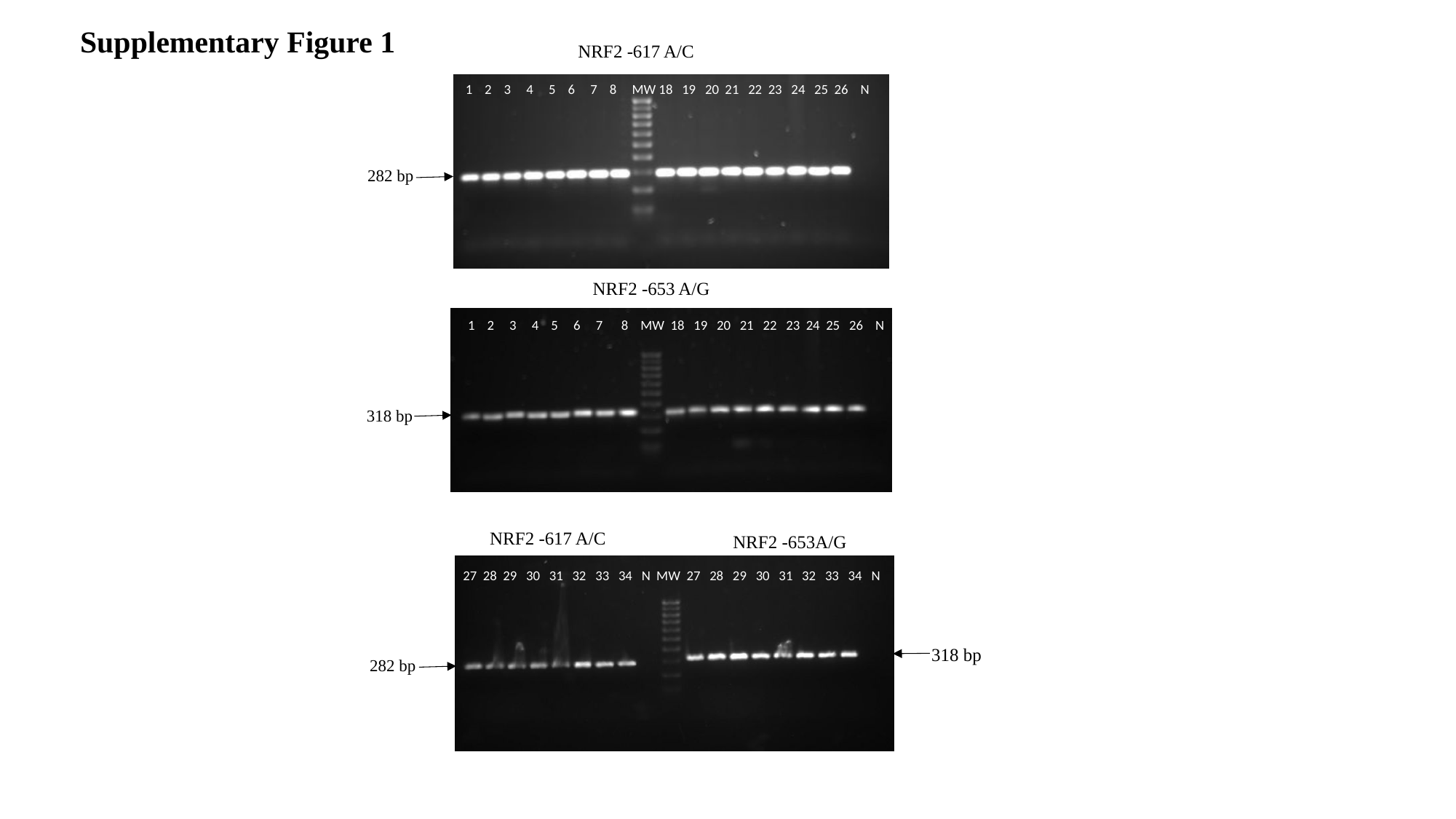

Supplementary Figure 1
NRF2 -617 A/C
1 2 3 4 5 6 7 8 MW 18 19 20 21 22 23 24 25 26 N
282 bp
NRF2 -653 A/G
1 2 3 4 5 6 7 8 MW 18 19 20 21 22 23 24 25 26 N
318 bp
NRF2 -617 A/C
NRF2 -653A/G
27 28 29 30 31 32 33 34 N MW 27 28 29 30 31 32 33 34 N
318 bp
282 bp

Supplement: Supplementary file 1 [file fsoa-05-361-s1.pptx]
